# Neutrophils and neutrophil extracellular traps in diabetes mellitus and its complications: Mechanisms and therapeutic implications

**DOI:** 10.1016/j.isci.2026.115585

**Published:** 2026-04-03

**Authors:** Li Lei, Jiaying Liu, Yunrong Li, Bo Huang, Zhenzhuang Zou

**Affiliations:** 1 Department of Pediatrics, Shenzhen Futian District Maternity & Child Healthcare Hospital, Shenzhen, Guangdong, China; 2 Pediatric Intensive Care Unit, The Third Affiliated Hospital of Zunyi Medical University, Zunyi, Guizhou, China; 3 Department of Pediatrics, The Affiliated Hospital of Zunyi Medical University, Zunyi, Guizhou, China

**Keywords:** health sciences

## Abstract

Diabetes mellitus is a chronic metabolic disease that has become a global health concern, leading to complications such as retinopathy, nephropathy, vasculopathy, and delayed wound healing. Neutrophils, as essential immune cells, protect the host by releasing neutrophil extracellular traps (NETs)—web-like structures composed of DNA and antimicrobial proteins that trap and kill pathogens. However, in diabetes, persistent hyperglycemia and inflammation induce excessive NET formation, resulting in endothelial damage, thrombosis, and tissue injury, which aggravate disease progression. Recent studies show that NETs not only participate in pathogen defense but also amplify chronic inflammation and vascular dysfunction in diabetic complications. This review highlights the dual role of neutrophils and NETs in diabetes, elucidates their molecular mechanisms in inflammation and vascular injury, and summarizes emerging therapeutic strategies targeting NETs—such as DNase I, PAD4 inhibitors, and IL-8 blockade—offering new insights for the prevention and treatment of diabetes and its complications.

## Graphical abstract

**Figure:**
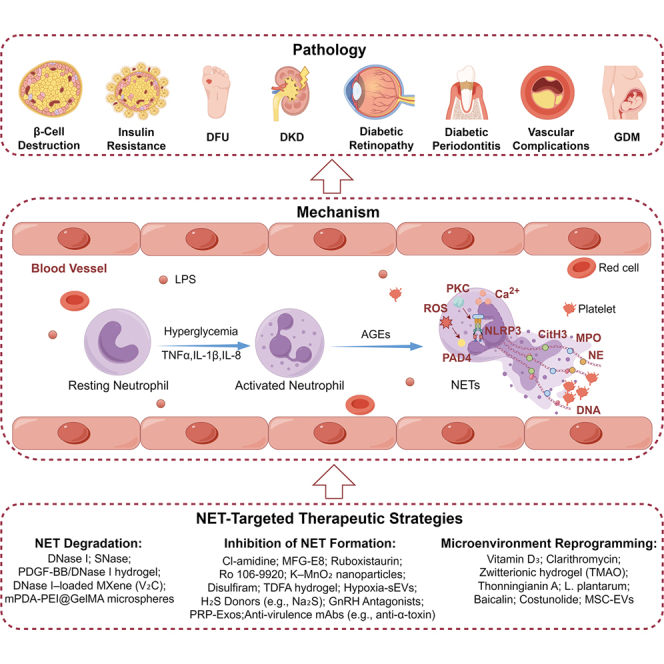

## Introduction

Diabetes mellitus (DM) represents one of the most pressing global public health challenges, with over 589 million individuals affected worldwide as of 2025, a number projected to rise to 853 million by 2050 (International Diabetes Federation, 2025).^1^ The impact of diabetes extends beyond hyperglycemia itself, with associated complications such as cardiovascular disease, diabetic kidney disease (DKD), and diabetic retinopathy (DR) contributing to significant morbidity and mortality.^2^ These facts underscore the systemic nature of diabetes, with long-term damage occurring across multiple organ systems. Recent studies have highlighted the role of neutrophil extracellular traps (NETs) in mediating pathogen defense in diabetes. Elevated NET formation in patients with diabetes contributes to an altered immune response, weakening pathogen clearance and leading to persistent infections.^3^ Furthermore, pathogens such as *Staphylococcus aureus* can evade NET-mediated defense by secreting DNases, further complicating immune responses and exacerbating disease progression in diabetes.^4^

Traditionally, diabetes has been conceptualized as a metabolic disorder primarily driven by insulin resistance and impaired insulin secretion. However, recent studies have underscored chronic low-grade inflammation, also referred to as metaflammation, as a key contributor to disease progression.^5^ Hyperglycemia induces oxidative stress, advanced glycation end-products (AGEs) accumulation, and amplifies immune responses through pathways such as TLR4-NF-κB and NLRP3 inflammasome activation, thereby exacerbating organ injury.^6^^,^^7^

Among the immune responses in diabetes, neutrophils, as essential effector cells of innate immunity, have a central role. In the diabetic milieu, persistent hyperglycemia alters neutrophil function, prolonging their lifespan, enhancing reactive oxygen species (ROS) production, and upregulating adhesion molecules, creating a primed state that predisposes them to tissue infiltration and damage.^8^^,^^9^^,^^10^^,^^11^ The discovery of NETs has added a new dimension to our understanding of neutrophil biology. NETs are web-like structures composed of decondensed DNA and granule proteins such as histones, myeloperoxidase (MPO), and neutrophil elastase (NE).^12^^,^^13^ Initially, NETs were thought to be an effective antimicrobial defense mechanism, but excessive or improperly cleared NETs can cause vascular endothelial injury, thrombosis, and autoimmune responses.^14^ In diabetes, NET formation is more easily triggered and can be reduced through improved glycemic control.^10^^,^^11^^,^^15^ Clinical studies have shown that NET-related biomarkers, such as extracellular DNA and MPO-DNA complexes, are elevated in the blood of patients with diabetes and are associated with the progression of renal and vascular complications.^16^^,^^17^ Accumulating evidence indicates that NETs represent a central mechanistic link between metabolic dysregulation and immune-mediated tissue injury in diabetes. Under hyperglycemic and chronic inflammatory conditions, excessive or dysregulated NET formation promotes endothelial damage, immune thrombosis, amplification of local inflammation, and impaired tissue repair, thereby driving the initiation and progression of diverse diabetic complications across organs.^18^^,^^19^

Importantly, this NET-driven pathogenic axis provides a unifying framework that links microvascular injury, sterile inflammation, immune thrombosis, and defective host-tissue interactions in diabetes. From a translational perspective, NET-associated pathways help explain why patients with apparently similar glycemic exposure can develop different complication profiles, and they also highlight NETs and their regulatory nodes as candidate biomarkers and therapeutic targets.^20^ This review therefore focuses not only on the biology of neutrophils and NETosis in diabetes, but also on how these mechanisms may inform risk stratification, complication-specific intervention, and the safer design of future NET-targeted therapies.

## Discovery of NETs and their structural features

The discovery of NETs has significantly advanced our understanding of innate immunity. In 2004, Brinkmann and colleagues reported that activated neutrophils release decondensed chromatin fibers decorated with granular and cytoplasmic proteins, forming web-like structures that trap and kill pathogens, including bacteria and fungi.^12^^,^^21^ This discovery unveiled a novel defense function of neutrophils beyond traditional phagocytosis and degranulation.

Initially, studies focused on the role of NETs in host defense against a wide range of pathogens, including parasites and viruses.^22^^,^^23^ However, this potent defense mechanism is not without its drawbacks. While NETs kill microbes and protect the host, some pathogens, such as *Streptococcus pneumoniae* and *Staphylococcus aureus*, can escape NET-mediated defense by producing DNases that degrade the DNA backbone.^24^^,^^25^^,^^26^ This “arms race” highlights the evolutionary significance of NETs and has inspired therapeutic strategies, including the use of exogenous DNase to mitigate NET-related damage.^27^

As research progressed, it became clear that NETs have roles far beyond pathogen defense. Around 2010, NETs were linked to sterile inflammation and autoimmune diseases, such as systemic lupus erythematosus (SLE) and rheumatoid arthritis (RA), where citrullinated histones act as autoantigens.^28^^,^^29^ Furthermore, studies later connected NETs with cancer, demonstrating that they promote tumor growth, metastasis, and cancer-related thrombosis.^30^^,^^31^^,^^32^ NET formation can also be induced by medical interventions, such as chemotherapy and mechanical ventilation, further highlighting their broad clinical relevance.^33^^,^^34^ As a result, NETs are now recognized not only as antimicrobial weapons but also as critical regulators of homeostasis and disease, with potential applications as biomarkers and therapeutic targets.

**Figure 1:**
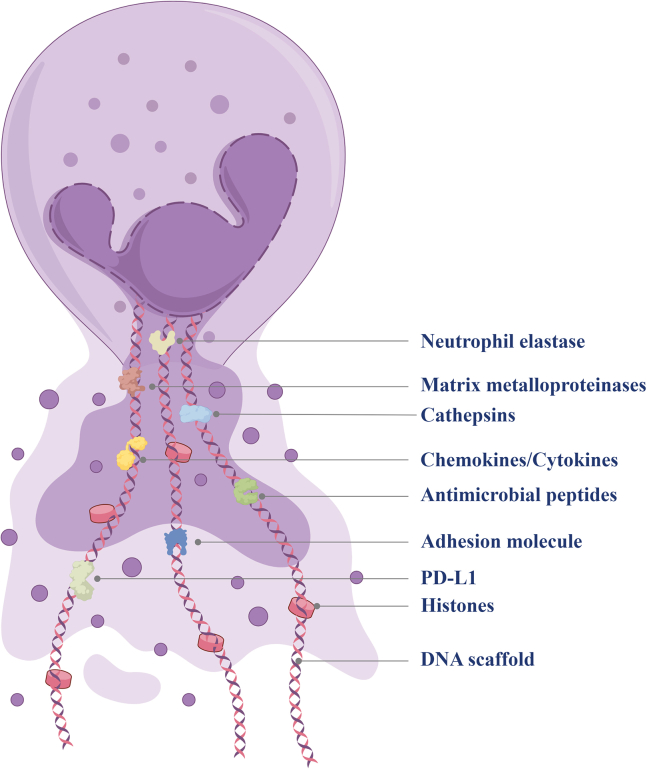
Structure and key components of neutrophil extracellular traps (NETs) NETs consist of a DNA scaffold associated with histones and various functional proteins, including neutrophil elastase, matrix metalloproteinases, cathepsins, chemokines/cytokines, antimicrobial peptides, adhesion molecules, and PD-L1.

The structure of NETs is fundamental to their function (Figure 1). NETs are extracellular, web-like structures composed of DNA filaments approximately 15–17 nm in diameter.^12^ The DNA backbone is primarily nuclear, although it can also originate from mitochondria. Mitochondrial DNA acts as a damage-associated molecular pattern (DAMP) and amplifies the inflammatory response.^35^^,^^36^ Extracellular DNA also possesses antimicrobial properties, such as binding essential metal ions, but it must be cleared by host DNases to prevent tissue damage.^37^

Histones (H1, H2A, H2B, H3, H4) constitute up to 70% of the proteins found in NETs.^38^ These proteins are not only structural components but also possess antimicrobial properties. However, histones can also be highly toxic and contribute to endothelial and tissue injury in diseases such as sepsis.^14^^,^^39^ Post-translational modifications of histones are critical for NET formation. Citrullination by PAD4 reduces their positive charge, weakens DNA binding, and promotes chromatin decondensation. NE further promotes chromatin expansion by cleaving histones.^29^^,^^40^^,^^41^

In addition to histones, NETs carry a wide range of granular and cytoplasmic proteins that shape both antimicrobial efficacy and host toxicity. MPO generates hypochlorous acid and strengthens oxidative killing, NE degrades microbial virulence factors and promotes chromatin expansion, and calprotectin restricts microbial growth by chelating zinc and manganese.^42^ Other components, including cathepsin G, proteinase 3, and defensins, broaden the antimicrobial repertoire of NETs while also increasing the potential for collateral tissue damage when NET clearance is insufficient.

NET formation can proceed through lytic (suicidal) or non-lytic (vital) pathways, but in both settings decondensed chromatin is externalized together with granular proteins to form a structure that is simultaneously protective and potentially injurious.^43^ This duality is central to the clinical significance of NETs in diabetes: The same scaffold that traps pathogens can also damage endothelium, promote thrombosis, and sustain sterile inflammation when NET production is excessive or resolution is delayed. Framing NETs in this way helps explain why they are increasingly viewed not only as antimicrobial effectors but also as biomarkers and therapeutic targets in chronic metabolic disease.

## Mechanisms of NET formation

### Activation of NETs

The formation of NETs is a multifaceted process that can be triggered by various stimuli through distinct pathways. The initial step involves the recognition of pathogen-associated molecular patterns (PAMPs) and DAMPs by specific receptors on neutrophils, such as TLRs, FcγRIIIb, CXCRs, C3aR, and RAGE, which subsequently activate downstream signaling cascades.^44^^,^^45^^,^^46^^,^^47^^,^^48^ Potent inducers of NET formation include bacteria such as *Streptococcus pneumoniae* and *Staphylococcus aureus*,^49^^,^^50^ viruses such as HIV, RSV, and SARS-CoV-2,^22^^,^^51^^,^^52^^,^^53^ as well as non-microbial stimuli such as autoantibodies, cytokines, tumor-derived factors, metabolites, and environmental particulates. Importantly, the aggregation state of pathogens, rather than their size, plays a critical role in the efficient activation of NET formation.^50^^,^^54^

### NET formation pathways

**Figure 2:**
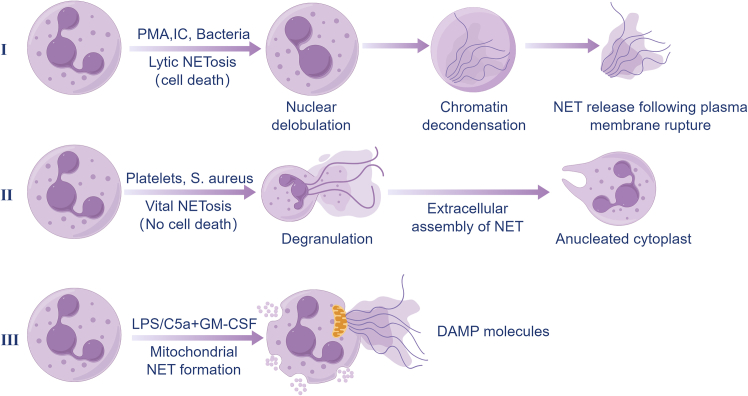
Major pathways of neutrophil extracellular trap (NET) formation Schematic illustration of the three major pathways of NET formation: lytic NETosis, characterized by chromatin decondensation and NET release following plasma membrane rupture; vital NETosis, involving extracellular NET release without cell death; and mitochondrial NET formation, in which mitochondrial DNA is released in response to specific stimuli.

In different pathological contexts, various mediators recruit neutrophils to the local microenvironment, where chemokine gradients guide their migration and promote NET formation.^44^ NETosis can be broadly classified into two main pathways: lytic (suicidal NETosis) and non-lytic (vital NETosis), with distinct mechanisms and functional consequences (Figure 2).

#### Lytic NETosis

Lytic NETosis is a form of programmed cell death characterized by NADPH oxidase (NOX)-dependent ROS generation.^55^^,^^56^ ROS drives the release of NE and MPO from azurophilic granules and enables their subsequent nuclear translocation. Within the nucleus, NE initiates partial histone degradation, loosening chromatin structure. MPO amplifies this process by generating HOCl, which in turn enhances NE-mediated proteolysis. This HOCl production not only promotes chromatin accessibility but also primes the chromatin for PAD4-mediated citrullination. Together, NE and MPO synergistically. This MPO-NE synergistic interaction creates permissive substrates for PAD4, which catalyzes histone citrullination and drives large-scale chromatin decondensation.^44^^,^^57^^,^^58^^,^^59^ In parallel, aberrant reactivation of cell cycle-associated kinases, particularly CDK4/6, acts as an upstream regulatory step by promoting nuclear envelope destabilization. This process facilitates nuclear permeability and enables subsequent chromatin expansion during lytic NETosis.^60^ Ultimately, the plasma membrane ruptures, releasing NETs after several hours.^56^^,^^61^

#### Non-lytic NETosis

In contrast, vital NETosis is a rapid (minutes), NOX-independent process that preserves neutrophil viability. In this context, NOX-derived ROS are indispensable for NET release.^61^^,^^62^ This pathway can be triggered by pathogens, such as *Candida albicans*, activated platelets, or complement components such as C5a via receptors such as TLR2 and C3.^43^^,^^47^^,^^54^ However, in specific settings, particularly during mtDNA extrusion, mitochondrial ROS may act as signaling modulators rather than primary drivers of chromatin decondensation. Importantly, this ROS involvement is mechanistically distinct from the NOX-dependent oxidative burst required for lytic NETosis.^43^^,^^63^

#### Clinical relevance and druggability of NET formation pathways in diabetic complications

While multiple signaling cascades, including ROS/NOX, PAD4, PKC, and NF-κB, regulate NET formation, their relevance to diabetic complications and translational potential is not equivalent. Recent genetic and pharmacological evidence supports a hierarchical organization of NET-associated pathways, with distinct importance across tissues and disease stages.^44^^,^^64^

The NOX-dependent ROS axis represents the most central and consistently validated driver of pathological NETosis in diabetes. Hyperglycemia and metabolic stress converge on NOX activation, leading to sustained ROS production that promotes NET formation across multiple diabetic complications, including retinopathy, kidney disease, thrombosis, and impaired wound healing. The inhibition of NOX-ROS signaling markedly reduces NET burden and tissue injury in experimental models, identifying this pathway as a primary upstream and druggable target.^65^^,^^66^^,^^67^^,^^68^

PAD4-mediated histone citrullination constitutes a secondary but highly druggable checkpoint, acting downstream of ROS to permit chromatin decondensation. PAD4 inhibition or genetic deletion effectively suppresses NET formation and improves pathological outcomes in inflammatory and autoimmune-dominant diabetic settings. However, its stimulus-dependent requirement suggests PAD4 targeting may be most effective in selected disease phenotypes rather than as a universal strategy.^44^^,^^69^^,^^70^

In contrast, PKC signaling, particularly PKCβ, functions mainly as a diabetes-specific amplifier of NETosis, enhancing ROS generation and endothelial dysfunction under chronic hyperglycemia. Although PKCβ inhibitors show clinical feasibility in diabetic microvascular disease, their effects on NETs appear largely indirect and tightly linked to metabolic control.^71^

Finally, NF-κB signaling acts as a contextual modulator, coupling NET formation to sustained inflammatory feedback loops. Given its broad immunoregulatory role, systemic NF-κB inhibition is unlikely to be optimal, favoring localized or combinatorial approaches.^72^^,^^73^

Collectively, these data support a hierarchical model of NET regulation in diabetic complications, in which ROS/NOX serves as the core metabolic driver, PAD4 as a chromatin-licensing checkpoint, and PKCβ and NF-κB as disease-specific amplifiers, providing a rational framework for prioritizing NET-targeted therapeutic strategies.

### Regulation of NETs

NETosis is a highly regulated process, with its magnitude, composition, and functional outcomes modulated at multiple levels.

#### Molecular regulation

The formation of NETs is orchestrated by tightly regulated molecular mechanisms. NE and MPO are indispensable effector enzymes for NET generation, and genetic deficiencies in these enzymes impair NET formation and compromise host defense.^74^^,^^75^^,^^76^ Additionally, post-translational modifications of histones, such as acetylation, further modulate the threshold for NET formation, allowing fine-tuning of NETosis.^77^ The enzyme PAD4 plays a critical role, and its function is stimulus-dependent. For instance, pharmacological inhibition of PAD4 effectively blocks nicotine-induced NETosis but not cholesterol crystal-induced NETosis, highlighting alternative activation pathways.^78^^,^^79^^,^^80^ Furthermore, the chromatin-binding protein DEK has emerged as a key regulator of NET formation. Its depletion significantly suppresses NET formation, while exogenous supplementation can rescue this defect.^81^^,^^82^

#### Neutrophil heterogeneity

Neutrophils are not a homogeneous population but rather consist of distinct functional subtypes. In healthy individuals, high-density neutrophils (HDNs) predominate in the circulation. In contrast, low-density neutrophils (LDNs) are enriched in pathological contexts such as SLE, antiphospholipid syndrome, and infections.^83^^,^^84^^,^^85^^,^^86^ LDNs typically display a pre-activated phenotype, characterized by enhanced spontaneous NET formation and immunosuppressive activity. Their abundance closely correlates with disease activity in autoimmune disorders, underscoring their pathogenic relevance.

#### Crosstalk and complex regulation

Lytic and non-lytic modes of NETosis are not mutually exclusive but exhibit significant cross-regulation. For example, following priming with lipopolysaccharide (LPS) or proinflammatory cytokines, mitochondrial DNA release—a hallmark of the non-lytic pathway—remains dependent on ROS signaling.^43^^,^^63^ Furthermore, certain stimuli, such as *Staphylococcus aureus*, may preferentially activate or simultaneously engage both pathways, depending on their aggregation state or the local microenvironment. These multilayered and interconnected regulatory circuits shape the functional outcomes of NETs and contribute to their pathological consequences.

## Strategies for detecting NETs

The detection of NETs presents significant challenges due to their complex structure, heterogeneous formation mechanisms, and rapid degradation *in vivo*. These factors contribute to the absence of a universally accepted “gold standard” detection method.^87^^,^^88^ Each available detection platform has distinct advantages and limitations, requiring researchers to carefully select appropriate methods based on the specific scientific question, sample type, and analytical goals. Often, combining multiple techniques is necessary for cross-validation.^89^

In practice, NET detection relies on complementary rather than competing methods. Morphological approaches remain the reference for confirming NET structures: Electron microscopy provides ultrastructural detail, whereas fluorescence or confocal microscopy combines DNA dyes with markers such as citrullinated histone H3 or MPO to distinguish NETosis from apoptosis and necrosis.^87^^,^^90^^,^^91^^,^^92^^,^^93^ Live-cell imaging further captures NET dynamics in real time and is particularly informative when mitochondrial ROS-dependent pathways are being interrogated.^94^

Solution-based assays and flow cytometry are especially relevant for translational and clinical studies because they permit higher-throughput analysis. ELISA-based detection of MPO-DNA or CitH3 complexes in plasma is widely used in cohort studies, but these readouts should be interpreted as surrogate markers rather than direct proof of NETosis because they may overlap with other forms of cell death or reflect differences in NET clearance.^43^^,^^95^^,^^96^^,^^97^ Flow cytometry and imaging flow cytometry add single-cell resolution and can improve objectivity, although suspension-based platforms may underestimate adhesion-dependent NETosis and may miss fully lysed structures.^98^^,^^99^^,^^100^^,^^101^^,^^102^

From a clinical standpoint, no single assay is currently sufficient for patient stratification or treatment monitoring in diabetes. The most informative strategy is to combine structural confirmation in experimental work with circulating biomarkers in clinical cohorts, while carefully accounting for infection, renal dysfunction, sampling conditions, and the tissue origin of extracellular DNA. Future progress will depend on standardized workflows, automated image analysis, and multimodal biomarker panels that can distinguish nuclear versus mitochondrial NET signatures and better link NET burden to specific diabetic phenotypes.^103^^,^^104^^,^^105^

## Hyperglycemia-driven NET formation: Pathological effects and interventions

The impact of hyperglycemia on neutrophils and NET formation represents a critical interface between metabolism and innate immunity. Importantly, hyperglycemia does not exert a uniform effect on NETosis. Instead, accumulating evidence indicates that its influence on NET formation is highly context-dependent, varying according to disease stage, inflammatory stimuli, tissue microenvironment, and metabolic adaptation. Under certain conditions, hyperglycemia markedly enhances NET formation, whereas in others it leads to functional dysregulation or attenuation of stimulus-induced NET responses.

**Figure 3:**
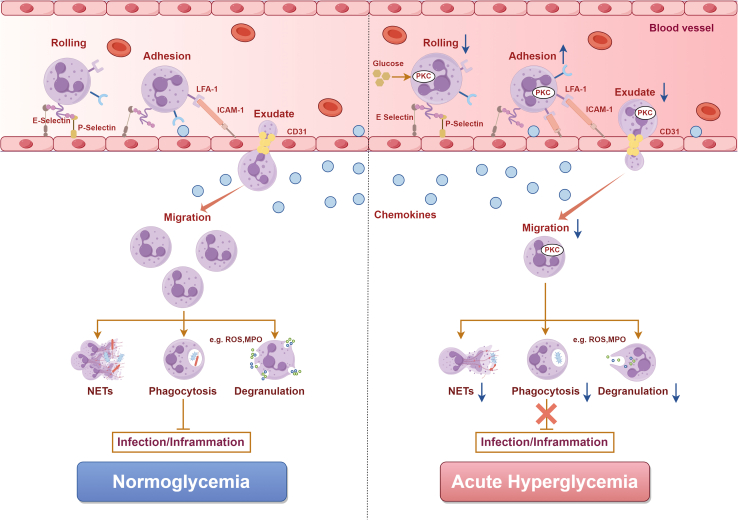
Hyperglycemia-induced impairment of neutrophil function Schematic illustration of the effects of acute hyperglycemia on neutrophil recruitment and effector functions. Compared with normoglycemia, acute hyperglycemia impairs rolling, adhesion, extravasation, and migration, and reduces NET formation, phagocytosis, and degranulation, thereby weakening host defense against infection and inflammation.

In acute or inflammation-associated hyperglycemic states, elevated glucose levels generally promote NET formation. Acute hyperglycemia activates PKC/MAPK and NF-κB signaling pathways, with TLR4 acting as a key metabolic-immune sensor, thereby amplifying innate immune activation and increasing susceptibility to inflammatory injury and infection.^106^ In parallel, hyperglycemia robustly activates the NOX-ROS axis, which serves as a central driver of NETosis, particularly in experimental models of DR^107^ (Figure 3). Consistent with this pro-NETotic phenotype, excessive NET formation is closely linked to β-cell autoimmunity and disease progression in type 1 diabetes (T1D).^14^

In contrast, during chronic hyperglycemia and prolonged metabolic stress, neutrophil function becomes reprogrammed rather than persistently hyperactivated. In type 2 diabetes (T2D), neutrophils frequently exhibit a “pre-activated” basal phenotype, but display attenuated IL-6–driven NET responses under hyperglycemic conditions.^108^ Moreover, metabolic reprogramming reduces neutrophil responsiveness to secondary inflammatory stimuli such as LPS, resulting in blunted stimulus-dependent NET formation despite the presence of chronic low-grade inflammation.^109^ These findings indicate that sustained metabolic stress can decouple NETosis from classical inflammatory signaling.

### Temporal dissociation and metabolic memory

Long-term clinical observations further highlight a temporal dissociation between glycemic control and NET burden. Even after effective normalization of blood glucose levels, circulating NET markers often remain elevated for prolonged periods and decline only gradually, typically over the course of approximately one year, in parallel with changes in systemic inflammatory markers.^15^^,^^110^

This delayed resolution suggests that hyperglycemia-induced NET formation may be influenced by metabolic memory, potentially mediated by persistent epigenetic remodeling or long-lasting post-translational modifications within neutrophils. Such temporal persistence provides a mechanistic bridge linking acute metabolic insults to chronic inflammatory complications in diabetes.^111^^,^^112^

### Unified pathological significance of NETs across tissues

Across diverse tissues, NETs act as amplifiers of chronic inflammation, barrier disruption, and impaired tissue repair under hyperglycemic conditions, thereby contributing to the progression of diabetic complications. In the vasculature, NETs damage the endothelial glycocalyx^113^ and exacerbate infarct size in ischemic stroke models.^114^ At barrier surfaces, excessive NET formation drives oral mucosal immunopathology^112^ and induces gut injury, which can be partially reversed by baicalin treatment^115^ and induces gut injury, which can be partially reversed by baicalin treatment.^116^ Mechanistically, hyperglycemia enhances NETosis through pathways involving GLUT1-mediated HMGB1 O-GlcNAcylation and TLR4 signaling,^117^ while NET accumulation also interferes with macrophage phenotype switching and resolution of inflammation.^118^

### Hierarchical organization of NET-targeted interventions

Therapeutic strategies targeting hyperglycemia-driven NET pathology can be broadly organized according to their primary mode of action. First, metabolic control-oriented interventions, such as metformin, reduce systemic glucose levels and concurrently correct NET-associated tissue dysfunction, including osteogenic defects.^116^ Second, nutrient-based and immunomodulatory interventions, exemplified by vitamin D_3_ supplementation, are associated with reduced NET formation in T2D populations and may modulate innate immune tone.^119^ Third, ROS- and NET-modulating natural compounds, including chalcones and baicalin, suppress oxidative bursts and attenuate NET-mediated tissue injury under hyperglycemic conditions.^115^^,^^120^ Finally, direct NET-targeting strategies, such as DNase I-mediated NET degradation and TLR2/4 antagonism, mitigate endothelial dysfunction and improve ischemic outcomes in animal and *in vitro* models of hyperglycemia.^114^^,^^121^

Collectively, these findings support a model in which hyperglycemia modulates NET formation through dynamic, context-specific mechanisms, involving oxidative stress, metabolic reprogramming, innate immune signaling, autophagy, and O-GlcNAc modification.^122^

Collectively, these findings support a model in which hyperglycemia modulates NET formation through dynamic, context-specific mechanisms, involving oxidative stress, metabolic reprogramming, innate immune signaling, autophagy, and O-GlcNAc modification.^102^ Strengthening glycemic control while selectively targeting NET formation or persistence may therefore represent a rational strategy for alleviating diabetic complications. Future studies should focus on the temporal dynamics of NETosis across disease stages, the molecular basis of metabolic memory, and the long-term safety of NET-targeted interventions, particularly with respect to host defense against infection.

## Neutrophils and neutrophil extracellular traps in diabetes and its complications

**Figure 4:**
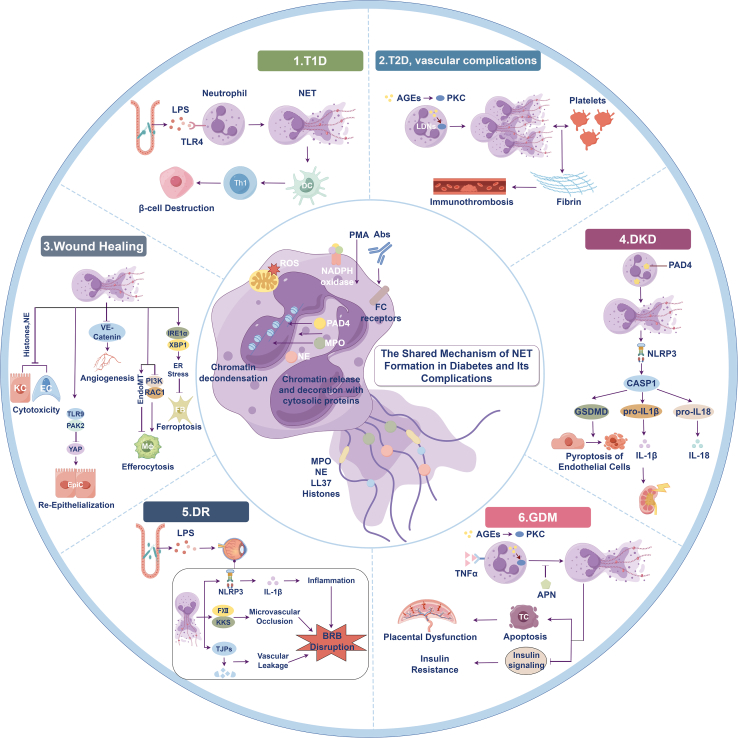
Roles of neutrophil extracellular traps (NETs) in diabetes mellitus and its complications Schematic illustration of the shared mechanisms of NET formation in diabetes and the contribution of NETs to the pathogenesis of type 1 diabetes, type 2 diabetes, vascular complications, diabetic kidney disease, impaired wound healing, diabetic retinopathy, and gestational diabetes mellitus.

Neutrophils and NETs play a critical and complex role in the development and progression of diabetes and its complications. Activated neutrophils and NETs contribute to the pathological processes associated with typical diabetes complications, including delayed wound healing, DR, DKD, and diabetic vascular complications (Table 1). In the unique hyperglycemic and inflammatory microenvironment of diabetes, dysregulated neutrophil function and aberrant NET formation may contribute to disease progression by promoting endothelial injury, exacerbating insulin resistance, and interfering with tissue repair processes. Mechanistic and translational studies further support these pathogenic roles of NETs across different target organs and vascular beds (Table 2; Figure 4).

**Table 1:** Clinical evidence of neutrophil extracellular traps (NETs) in patients with diabetes

Disease type	Study design and population	NET-related markers	Main clinical findings	Clinical relevance	Reference
Type 1 diabetes	Case-control; newly diagnosed T1D	NE, PR3, MPO-DNA, CitH3	NET markers were significantly elevated at disease onset and correlated with autoantibody titers	NETs participate in early autoimmune activation	Wang et al.^44^
Type 1 diabetes	Cross-sectional; established patients with T1D	cfDNA, MPO-DNA	NET levels remained elevated despite glycemic control	Supports inflammation-related metabolic memory	Klocperk et al.^123^
Type 2 diabetes	Case-control; patients with T2D vs. controls	NET formation capacity, ROS	Neutrophils were primed for NETosis but showed impaired bactericidal activity	Explains increased infection susceptibility	Farhan et al.^124^
Type 2 diabetes	Cohort; patients with T2D with/without thrombosis	MPO-DNA, cfDNA	Increased NET burden associated with a prothrombotic, hypofibrinolytic phenotype	NETs drive immunothrombosis	Bryk et al.^65^
Type 2 diabetes	Case-control; patients with T2D	CitH3, MPO	NET markers correlated with microvascular injury	NETs as potential biomarkers of vascular damage	Menegazzo et al.^11^

**Table 2:** Clinical studies linking NETs to diabetic complications

Complication	Patient cohort	Sample	NET markers	Key clinical observations	Prognostic/translational implication	Reference
Diabetic foot ulcer	Patients with T2D with DFUs	Wound tissue, plasma	CitH3, MPO-DNA	Excessive NET accumulation in wounds associated with delayed healing	NET burden predicts poor wound outcome	Ibrahim et al.^125^
Diabetic foot ulcer	Patients with DFU	Wound biopsy	PAD4, CitH3	PAD4-driven NETosis enriched in non-healing wounds	PAD4 as a therapeutic target	Wong et al.^10^
Diabetic retinopathy	Patients with T2D with DR	Plasma	MPO, CitH3	Elevated circulating NET-associated markers in diabetic retinopathy, with higher levels reported in advanced stages	NET burden may reflect disease severity and inflammatory progression	Magaña-Guerrero et al.^126^
Diabetic kidney disease	Patients with biopsy-confirmed DKD	Kidney tissue, plasma	CitH3, MPO	NET deposition on glomerular capillaries	NETs contribute to renal inflammation and injury	Zheng et al.^127^
Diabetic vascular complications	Patients with T2D with cardiovascular events	Plasma	MPO-DNA, cfDNA	NETs associated with hypofibrinolytic thrombosis	Supports NET-targeted antithrombotic strategies	Bryk et al.^65^

### Diabetes

NETs play a crucial role in diabetes, with clear differences in their involvement across disease types, stages, and tissues.^70^^,^^128^ NETs primarily drive disease progression by mediating chronic inflammation, immune thrombosis, and endothelial damage. However, their specific mechanisms and clinical significance differ between T1D and T2D. Although circulating NET-associated biomarkers, such as cell-free DNA (cfDNA) and myeloperoxidase-DNA (MPO-DNA) complexes, are consistently elevated in patients with diabetes, their clinical interpretation remains limited. cfDNA is highly sensitive but lacks specificity, as it may derive from multiple forms of cell death and is strongly influenced by renal dysfunction, infection, and tissue injury—conditions frequently present in diabetic populations.^14^^,^^129^ In contrast, MPO-DNA complexes provide greater specificity for NET formation and are therefore more widely accepted as surrogate markers of NETosis; however, their application is constrained by assay variability, lack of methodological standardization, and limited comparability across studies, particularly in heterogeneous clinical cohorts.^95^^,^^130^ Moreover, most available clinical studies are cross-sectional or involve relatively small sample sizes, which restricts causal inference and limits their utility for disease staging, prognostic assessment, and therapeutic monitoring.^131^ Collectively, these limitations highlight the need for combinatorial biomarker strategies and longitudinal validation in well-phenotyped diabetic cohorts to more robustly define the association between NET levels, disease severity, and clinical outcomes, and to enhance translational relevance. Although both T1D and T2D are associated with elevated NET burden, the biological context, functional consequences, and clinical implications of NET formation differ substantially between autoimmune-driven T1D and metabolically driven T2D.

In T1D, NETs primarily contribute to the initiation and amplification of autoimmune responses, reflecting an inflammation-dominated and immune-driven disease context. Clinical studies demonstrate that in newly diagnosed patients with T1D, circulating NET-associated markers, including NE, PR3, and CitH3, are significantly elevated and positively correlate with autoantibody levels.^16^^,^^132^^,^^133^^,^^134^ , supporting a role for NETs in early disease activity. Histopathological analyses further reveal neutrophil infiltration and NET deposition in pancreatic tissue at preclinical stages, accompanied by a reduction in peripheral neutrophil counts.^135^^,^^136^^,^^137^ Mechanistically, NET formation in T1D is closely linked to intestinal barrier disruption and microbial translocation. Gut-derived microbial products, such as LPS, activate neutrophils via the TLR4/ROS/PAD4 signaling axis, promoting NET release.^132^^,^^138^^,^^139^ NET components can subsequently cross-react with insulin antigens, activate dendritic cells, and enhance Th1-skewed immune responses, thereby accelerating pancreatic β-cell destruction.^140^ Platelet-neutrophil aggregates further amplify these autoimmune and inflammatory cascades.^141^^,^^142^ Despite this heightened NET burden, neutrophils in T1D display impaired antimicrobial functions, including defective chemotaxis, phagocytosis, and regulated NET deployment.^143^^,^^144^ Importantly, excessive NET formation should not be equated with enhanced host defense. Instead, NET overproduction and immune insufficiency represent dissociable yet coexisting aspects of neutrophil dysfunction in T1D. Consistent with this complexity, circulating NET markers vary substantially across disease stages, individuals, and clinical subtypes.^142^^,^^145^^,^^146^ Proteomic profiling further indicates that T1D-associated NETs are enriched in metabolic-related proteins, underscoring disease-specific functional reprogramming rather than uniform antimicrobial activation.^147^^,^^148^

In contrast to the autoimmune-driven context of T1D, NET formation in T2D occurs against a background of chronic metabolic dysregulation and low-grade inflammation. The disruption of the immune-metabolic axis, driven by glucolipotoxicity, advanced glycation end (AGEs) products, and PKC activation, induces metabolic reprogramming of neutrophils and promotes NETosis-associated functional defects.^69^^,^^144^^,^^148^ These alterations contribute to impaired tissue repair and increased susceptibility to infection. NETs in T2D also play a central role in vascular pathology. By reinforcing fibrin clot architecture and resisting fibrinolysis, NETs promote atherosclerotic progression and thrombotic complications.^128^^,^^149^ Circulating NET levels correlate with the severity of vascular damage and smoking intensity, linking NET burden to clinical disease severity.^150^ LDNs, which expand in T2D, exhibit a pronounced NETotic phenotype^151^^,^^152^ and can drive excessive inflammatory responses during infection through platelet activation.^153^ Metabolic mediators such as homocysteine further enhance NET formation via NOX- and calcium-dependent signaling pathways, establishing a positive feedback loop with IL-6.^154^ Proteomic analyses of neutrophils and NETs from patients with T2D reveal characteristic signatures of metabolic and inflammatory reprogramming, including enhanced glycolytic flux and downregulation of immune defense proteins.^139^^,^^144^ These findings support a model in which NET formation is quantitatively increased but functionally uncoupled from effective antimicrobial responses, rather than acting as a direct causal driver of host defense. Complementary bioinformatic studies have identified diagnostic gene signatures associated with a T2D-NET score,^155^ and clinical investigations demonstrate that neutrophils with a high inflammatory phenotype are linked to adverse outcomes following acute coronary syndrome in patients with diabetes.^156^

Mechanistically, NET formation in both T1D and T2D shares a core pathway: hyperglycemia/inflammation → ROS/NOX → PAD4-mediated histone citrullination → NET release, with platelet-neutrophil interactions and immune thrombosis amplifying the process.^69^^,^^142^ Based on these mechanisms, targeting NETs has emerged as a promising therapeutic strategy. Preclinical studies demonstrate that interventions such as DNase I and PAD4 inhibitors can improve the phenotypes and reduce the risk of diabetes complications.^3^^,^^157^ Drugs such as metformin,^134^^,^^158^ liraglutide,^3^^,^^152^ irisin,^159^ and diethylcarbamazine^160^ have also been found to inhibit NET formation. Although NETs play a crucial role in the pathogenesis of diabetes, most of these findings come from preclinical research, and their clinical translation remains challenging. Issues such as inconsistency in circulating biomarkers, disease-stage specificity, and the paradox between NET formation and function are critical obstacles that need to be addressed. Future research should focus on standardizing detection methods, conducting longitudinal studies in stratified patient populations, and rigorously evaluating NET-targeted interventions. Clinically, this means that NET assessment in diabetes is likely to be most informative when interpreted in relation to disease subtype, inflammatory activity, infection burden, and vascular phenotype rather than as a standalone universal biomarker.

### Diabetic wound healing

Excessive formation of NETs has emerged as a critical driver of impaired tissue repair in chronic diabetic wounds, particularly diabetic foot ulcers (DFUs).^10^^,^^161^ In the complex microenvironment of hyperglycemia, insulin resistance, and associated metabolic dysregulation, neutrophils acquire a “pre-activated” phenotype, characterized by delayed early recruitment and prolonged infiltration. This is accompanied by the upregulation of PAD4 and an explosive release of ROS, driving citH3 and excessive NET formation.^10^^,^^162^^,^^163^^,^^164^^,^^165^ Both animal models and clinical studies consistently demonstrate that the levels of NET markers (such as citH3, MPO-DNA complexes, and cfDNA) in wound tissue and circulation correlate with delayed healing and increased risk of amputation.^164^^,^^166^^,^^167^^,^^168^^,^^169^ Notably, exogenous factors such as nicotine and key therapeutic agents such as insulin have been shown to bidirectionally regulate NETosis.^162^^,^^163^ Emerging evidence further reveals an intersection between NETs and ferroptosis, a newly identified regulated cell death pathway, highlighting the profound metabolic reprogramming that influences neutrophil immune function and their interaction with other forms of cell death.^169^

Mechanistic studies reveal that NETs disrupt tissue repair through a networked mechanism. The physical structure and protein components of NETs, such as histones and elastase, directly damage keratinocytes and endothelial cells. Additionally, NETs activate multiple signaling axes, including TLR4/TLR9-NF-κB and TLR9-PAK2–Hippo-YAP, which induce NLRP3 inflammasome activation and endothelial-to-mesenchymal transition, thereby severely impairing reepithelialization, angiogenesis, and sustaining chronic inflammation.^164^^,^^169^^,^^170^^,^^171^ Concurrently, interactions between NETs and fibroblasts form a vicious cycle: NETs promote extracellular matrix degradation through an IL-8/MMP-9 autocrine loop,^172^ and trigger IRE1α/XBP1-mediated endoplasmic reticulum (ER) stress, inducing ferroptosis in fibroblasts, further hindering tissue remodeling.^169^^,^^173^ In immune regulation, excessive NETs inhibit macrophage efferocytosis by suppressing the PI3K/Rac1 pathway, leading to persistent inflammation. Moreover, the LRG1–TGFβ/ALK5–Akt positive regulation and TREM1–FOXM1 negative regulation finely modulate the threshold for NETosis.^164^^,^^166^^,^^174^^,^^175^ However, how these highly refined pathways are integrated and prioritized within the complex DFU environment, which simultaneously involves ischemia, infection, and metabolic abnormalities, remains an unresolved question.

From a translational perspective, the exploration of NETs markers in plasma or tissue (e.g., NLR, PLR, citH3, and cfDNA) shows promise for predicting wound prognosis.^164^^,^^165^^,^^167^^,^^168^ However, challenges remain, as markers such as cfDNA lack specificity and are susceptible to degradation by bacterial DNases. Despite advancements in rapid quantification techniques, such as one-step qPCR, improving detection sensitivity,^176^ the standardization and clinical feasibility of these techniques in multicenter cohorts require further validation. Therapeutically, targeting NET formation (e.g., PAD4 inhibitors such as Cl-amidine), NET clearance (e.g., DNase I), or downstream toxicities (e.g., anti-histone antibodies), as well as intervening in related pathways (e.g., using disulfiram to inhibit NLRP3/GSDMD, ferroptosis inhibitors such as ferrostatin-1, or CXCR1/2 antagonists such as reparixin) have shown efficacy in preclinical models.^10^^,^^164^^,^^165^^,^^169^^,^^170^^,^^176^ However, broad suppression of NETs could undermine host defense against infections, and the lack of selectivity in some inhibitors may lead to off-target effects. A more promising approach is the development of spatially and temporally precise interventions, such as localized drug delivery using smart hydrogels or nanomaterials (e.g., IRE1α inhibitors^173^ or PI3K/Rac1 activators^175^), exploring combined targeting of the LRG1/TREM1-FOXM1 axis,^164^^,^^166^^,^^174^ optimizing metabolic control (e.g., precision insulin use^163^), and integrating novel approaches such as stem cell therapy and biomaterial-based strategies. These efforts aim to reshape the wound healing microenvironment while preserving host defense functions. Accordingly, DFUs may represent the most immediately translatable setting for NET-targeted therapy because local delivery can be combined with debridement, infection control, and vascular assessment while minimizing systemic immunosuppression.

### Diabetic retinopathy

DR is the most common microvascular complication of diabetes and is increasingly recognized as an inflammation-driven disease.^177^^,^^178^^,^^179^ Recent studies have highlighted a key role for NET-driven inflammation in compromising retinal vascular integrity. NET-related markers, such as citH3, MPO, and NE, are consistently elevated in the circulation of patients with DR.^180^^,^^181^^,^^182^ Hyperglycemia has been shown to induce NET formation through NOX-dependent ROS production and PAD4-mediated histone citrullination, a mechanism common across diabetic complications and implicated in DR.^107^^,^^183^^,^^184^ Although the core mechanisms of NET formation are shared across diabetic complications, transcriptomic and Mendelian randomization analyses have identified NET-related genes—such as ALOX5, CXCL5, and OSM—as being preferentially associated with DR. These findings support a context-specific contribution of NET-associated pathways to retinal pathology within the unique microvascular and inflammatory environment of the retina, rather than implying absolute disease specificity.^185^

At the retinal level, NETs disrupt vascular homeostasis through multiple mechanisms. NE and citrullinated histones degrade tight junction proteins such as VE-cadherin and occludin, weakening the inner blood-retina barrier and promoting vascular leakage.^183^^,^^186^ Additionally, NET-derived DNA and serine proteases activate leukocyte adhesion molecules and coagulation pathways, including the kallikrein-kinin cascade and factor XII activation, further fueling local inflammation and microvascular occlusion.^183^^,^^187^ These events collectively contribute to the hallmark features of advanced DR, including ischemia, capillary dropout, and neovascularization. Therapeutically, disrupting NET formation or enhancing NET degradation (e.g., using PAD4 inhibitors or DNase I) has been shown to alleviate retinal leakage and inflammation in experimental models.^183^^,^^188^

These pronounced local effects of NETs on retinal vascular integrity have prompted further investigation into whether NET-driven inflammation in DR is confined to the retina or reflects a broader systemic process. Beyond the retina, recent studies have revealed a systemic component to NET involvement in DR. In murine models of T2D, NETosis occurs in the intestinal mucosa, where it disrupts epithelial barriers and facilitates the translocation of microbial products such as LPS and PGN into the bloodstream.^188^ These circulating PAMPs activate TLR and NOD pathways, which in turn promote retinal inflammation and secondary NET deposition.^188^^,^^189^ Thus, NETs may serve as intermediaries linking gut-derived immune dysregulation to retinal injury. Altogether, these findings support a model in which NETs act both as local disruptors of vascular integrity and as systemic amplifiers of inflammation, integrating metabolic, immunologic, and barrier-related dysfunctions in the pathogenesis of DR. These observations suggest that in DR, NET-related biomarkers may be most useful as adjuncts to stage disease activity and to identify patients in whom local barrier-protective or anti-inflammatory strategies deserve further testing alongside established ophthalmic care.

### Diabetic kidney disease

DKD remains the leading cause of kidney failure worldwide. Despite the availability of therapies such as renin-angiotensin-aldosterone system inhibitors and sodium-glucose cotransporter 2 inhibitors, preventing kidney failure and cardiovascular events in patients with diabetes continues to be a major clinical challenge.^190^^,^^191^^,^^192^ Increasing evidence suggests that DKD is not only a metabolic disorder but also an immune-driven disease, with chronic inflammation and immune cell activation playing central roles.^193^^,^^194^ In hyperglycemic conditions, neutrophils release NETs, which have been observed both *in vitro* and in patients with DKD.^195^^,^^196^ PAD4-dependent NET deposition on glomerular capillaries has been demonstrated in human kidney biopsies, indicating a pathogenic role in glomerular injury.^195^

Mechanistically, NETs exacerbate DKD progression by activating the NLRP3 inflammasome, leading to caspase-1–dependent cleavage of gasdermin D and pro-IL-1β, thereby inducing endothelial pyroptosis and the maturation and release of IL-1β,^196^ and promoting tubular injury through CASP1- and LYZ-mediated inflammation.^197^ Bioinformatic analyses and experimental validation have further identified NET-related genes, such as ITGAM, ITGB2, and IL-33, which correlate with immune activation and adverse clinical outcomes in DKD.^197^^,^^198^ Among these, IL-33 appears to be particularly important, as it amplifies neutrophil activation via the IL-33–ST2 axis, sustaining NET formation.^198^

However, discrepancies in the literature remain. While several studies confirm excessive NET deposition in diabetic kidneys,^195^^,^^196^^,^^199^ some murine studies suggest that NET formation occurs only in the presence of overt inflammation, rather than hyperglycemia alone.^200^ Moreover, despite heightened NET activity in DKD, neutrophils from individuals with diabetes have also been reported to show impaired NET formation in response to classical inducers, such as LPS or PMA, potentially increasing susceptibility to infections.^201^ These inconsistencies are likely due to differences in disease stage, immune context, or experimental design. Overall, NETs appear to act as a double-edged sword in DKD—contributing to sterile inflammation and tissue injury while potentially impairing antimicrobial defense. Clarifying these dual roles, dissecting stage-specific mechanisms, and testing targeted interventions, such as PAD4 inhibition, DNase therapy, or IL-33 blockade, are critical for advancing our understanding of DKD and developing effective therapeutic strategies.

### Diabetic periodontitis

Patients with diabetes are at an increased risk for periodontitis, with both the severity and progression of the disease being greater than in non-diabetic individuals.^202^^,^^203^ Hyperglycemia plays a significant role in the development of periodontitis by promoting NET formation. Elevated blood glucose enhances neutrophil activation, which, in turn, leads to excessive NET release. This process suppresses macrophage JAK2/STAT3 signaling, drives M1 polarization, and impairs M2 repair mechanisms, amplifying inflammation and accelerating alveolar bone loss.^204^ This makes the oral cavity another plausible site for locally delivered NET-modulating strategies, although current evidence remains preliminary.

### Diabetic vascular complications

In diabetic vascular complications, NETs are emerging as key mediators that link inflammation, metabolic dysregulation, and thrombosis. Clinical studies have demonstrated elevated NET markers in patients with T2DM, which correlate with hypofibrinolysis and the formation of dense fibrin networks.^65^ In contrast, such associations are less consistent in long-standing T1DM, suggesting that the duration of the disease and therapeutic regimens may influence NET levels.^205^^,^^206^

Mechanistic investigations have shown that cardiac metabolic stress promotes NET formation through the PDK4-lactate-PRMT9-PFKL axis.^206^ The gut-derived metabolite phenylacetylglutamine has also been shown to enhance NET generation and exacerbate ischemic injury in myocardial infarction and stroke.^207^ Collectively, NETs not only function as effector molecules in diabetic vascular complications^208^ but also serve as a mechanistic link between metabolic abnormalities and a prothrombotic phenotype. Nevertheless, much of the current evidence comes from cross-sectional studies and experimental models, and the causal role of NETs requires further validation through prospective studies and interventional trials.^209^^,^^210^ In clinical practice, the greatest promise may lie in using NET-related markers to refine thromboinflammatory risk rather than immediately adopting broad systemic NET inhibition, which could increase infection risk in high-risk diabetic populations.

### Diabetic hepatocellular carcinoma

Patients with diabetic hepatocellular carcinoma (HCC) are more prone to postoperative recurrence and metastasis, with significantly higher mortality rates than patients with non-diabetic HCC.^211^^,^^212^^,^^213^ Compared to their non-diabetic counterparts, patients with diabetic HCC exhibit elevated levels of NETs and reduced expression of DNASE1L3 in both serum and tumor tissues. Similar results have been observed in diabetic mouse models of HCC, where excessive NET accumulation promotes tumor invasion and metastasis. This effect is likely due to DNASE1L3 deficiency, which leads to NET DNA accumulation and subsequent activation of the cGAS-noncanonical NF-κB pathway, thereby upregulating MMP9 and SPP1.^214^

### Diabetic erectile dysfunction

In the STZ-induced diabetic rat model, excessive NET formation triggers the activation of the NLRP3 inflammasome, leading to the pyroptosis of cavernosal smooth muscle cells and local inflammation. This results in tissue damage and erectile dysfunction. However, the use of DNase I to degrade NETs has been shown to improve this condition.^215^

### Gestational diabetes mellitus

Gestational diabetes mellitus (GDM) is a multifactorial disorder involving metabolic, immune, and social factors. The pathophysiology of GDM includes β-cell dysfunction, adipose tissue imbalance, placental maladaptation, and epigenetic reprogramming, all contributing to long-term health risks for both mothers and offspring.^216^^,^^217^^,^^218^ In recent years, the abnormal activation of NETs has emerged as a central link between immune dysregulation and metabolic disturbance, providing a new paradigm for understanding GDM.

Studies have shown that high blood sugar and inflammatory factors (e.g., TNF-α) together promote NET formation in GDM. This process may be driven by the accumulation of ROS and the activation of NOX.^219^ Additionally, reduced adiponectin levels in patients with GDM further aggravate abnormal NET formation, worsening disease progression. Mechanistically, NETs have been implicated in insulin resistance through interference with insulin signaling pathways and induction of local inflammatory responses.^220^ However, the causal hierarchy of these interactions remains incompletely defined.^221^

Recent studies have suggested the therapeutic potential of targeting NETs in GDM. In mouse models, (TMAO) has been shown to reduce NET formation and improve placental function and fetal development. Notably, whether the metabolic effects of TMAO on insulin sensitivity are directly mediated through NET modulation or reflect parallel regulatory pathways remains to be fully elucidated.^222^ While these findings provide preliminary evidence for clinical translation, further *in vivo* and *in vitro* studies, as well as safety evaluations, are needed. Given the maternal-fetal context, any NET-targeted strategy in gestational diabetes will require an especially high safety threshold, making mechanistic clarification and local or short-duration interventions more attractive than chronic systemic immunomodulation.

## Neutrophil-targeting therapies for diabetes and its complications

Therapeutic approaches aimed at mitigating NET-driven pathology in diabetes have expanded rapidly, encompassing direct enzymatic degradation of NETs, pharmacological blockade of NET formation, immune modulation, and advanced biomaterial-based delivery systems (Table 3). Notably, many of these strategies have been independently explored by multiple research groups across diverse diabetic models, providing growing preclinical evidence base for translational development. Therapeutic approaches aimed at mitigating NET-driven pathology in diabetes have expanded rapidly, encompassing direct enzymatic degradation of NETs, pharmacological blockade of NET formation, immune modulation, and advanced biomaterial-based delivery systems (Table 1). The optimal therapeutic choice is likely to depend on the specific diabetic complication and must be balanced against the indispensable role of neutrophils in host defense. These considerations underscore a precision-medicine paradigm: attenuating pathological NET activity without eroding antimicrobial competence.^224^^,^^225^

**Table 3:** Potential neutrophil-associated therapeutic targets in diabetes and its complications

Target	Drugs/Agents	Observed effects/application status	Reference
NET degradation	DNase I	Promotes corneal and nerve regeneration in diabetic mice; limited by short half-life; modest efficacy in lupus nephritis, suggesting resistance of chronic NETs (preclinical/limited clinical use)	Jin et al.^143^; Thimmappa et al.^144^
NET degradation	Staphylococcal nuclease (SNase, via L. lactis)	Degrades intestinal NETs, improves barrier, delays T1D onset in NOD mice (preclinical)	Qin et al.^145^
PAD4	Cl-amidine	Delays T1D onset, reduces autoantibody titers, improves gut barrier; inhibits NETosis (preclinical)	Bissenova et al.^146^
NLRP3 inflammasome	Disulfiram (free or hydrogel)	Inhibits NLRP3/caspase-1/GSDMD-driven NETosis; accelerates wound healing; hydrogel enhances M2 polarization and local delivery (preclinical/translational)	Bissenova et al.^147^; Yaw et al.^148^
PKCβ	Ruboxistaurin	Promotes angiogenesis, restrains NETosis, accelerates wound closure; potential in retinopathy and wounds (clinical trials in DR; exploratory for NETs)	Zhu et al.^69^
Virulence factor (α-toxin)	mAb against S. aureus α-toxin	Suppresses toxin-driven NETosis, preserves tissue, improves healing in infected wounds (preclinical)	de Vries et al.^149^
GnRH receptor	GnRH antagonists	Suppress PAD4-mediated NET formation; improve wound healing; receptor upregulated in DFUs (preclinical/human tissue evidence)	Chatzigeorgiou et al.^150^
ROS/MAPK-PAD4 axis	H_2_S donors (Na_2_S)	Scavenge ROS, block MAPK pathway, suppress NETosis, enhance repair (preclinical)	Dumont et al.^151^
NF-κB	Ro 106-9920	Downregulates PAD4; interrupts NET-NLRP3 loop; reduces inflammation and restores repair function (preclinical)	Zhong et al.^152^
Micronutrient	Vitamin D_3_	Inhibits NET formation in *ex vivo* T2D neutrophils; dietary adjunct potential (*ex vivo* human evidence)	Cohen et al.^153^
Antibiotic (macrolide)	Clarithromycin	Enhances NET antimicrobial and pro-healing function (LL-37 loading) without full suppression; improves fibroblast migration and collagen deposition (clinical/translational)	Joshi et al.^154^
Natural compounds	Baicalin	Mitigates hyperglycemia-induced gut barrier impairment via NET inhibition (preclinical)	He et al.^155^
Natural compounds	Costunolide	Reduces renal inflammation and thrombosis in diabetic nephropathy via NET suppression (preclinical)	Barbu et al.^156^
Natural compounds	Thonningianin A (hydrogel)	Accelerates wound healing via antimicrobial effect and M1→M2 polarization (preclinical/biomaterial-based)	Lang et al.^157^
Probiotics	L. plantarum	Improves DFU healing by modulating microbiota, angiogenesis, and neutrophil activity (clinical exploratory)	Shafqat et al.^3^
Ca^2+^ signaling	K-MnO_2_ nanoparticles	Recalibrate Ca^2+^ signaling, restore phagocytosis, improve healing in MRSA-infected wounds (preclinical/nanotherapy)	Thierry^158^
NET clearance/reprogramming	DNase I-loaded MXene (V_2_C)	Shifts neutrophils from NETosis to phagocytosis; disrupts biofilms; clears infection (preclinical)	Tan et al.^159^
PAD4/immune microenvironment	TDFA hydrogel	Suppresses NETs, promotes macrophage polarization, and fibroblast migration. (preclinical/local delivery)	Segoviano-Ramirez et al.^160^
Sequential release	PDGF-BB/DNase I hydrogel	Recruits cells, then degrades NETs; synergistically enhances healing (preclinical)	Fadini et al.^161^
Immunomodulatory biomaterial	Zwitterionic hydrogel (TMAO)	Suppresses excessive NETs, promotes regeneration by microenvironment modulation (preclinical)	Aspera-Werz et al.^162^
NET scavenging	mPDA-PEI@GelMA microspheres	Bind and neutralize NETs without pathogen release (preclinical)	Linnemann et al.^163^
MSC-derived EVs	MSC-EVs	Prevent NET-induced ferroptosis in endothelial cells; promote angiogenesis and healing (preclinical/translational)	Ma et al.^164^
Preconditioned EVs	Hypoxia-sEVs	Deliver miR-17-5p; suppress TLR4/ROS/MAPK axis; reduce NETs and ER stress (preclinical)	Nambi et al.^223^
PRP-derived exosomes	PRP-Exos	Deliver miR-26b-5p; inhibit MMP-8–driven NETosis; improve wound healing (preclinical/autologous translational)	Liu et al.^166^
Endogenous immunoregulator	MFG-E8	Breaks NET-NLRP3 loop (suppresses IL-1β-NETs, blocks P2X7); reduces inflammation and accelerates repair (preclinical)	Yang et al.^167^

### Enzymatic degradation of NETs

One of the most direct strategies is to supplement exogenous nucleases when endogenous DNase activity is impaired.

#### DNase I

Topical DNase I promotes corneal epithelial and nerve regeneration in diabetic mice by clearing cytotoxic NETs, reducing inflammation, and re-activating pro-healing signaling.^226^ However, its translational utility is constrained by a short half-life and the operational burden of repeated dosing. Moreover, the suboptimal responses to recombinant human DNase I in lupus nephritis^227^ suggest that NETs formed under chronic disease conditions may develop nuclease resistance, posing a challenge for diabetes.

#### Staphylococcal nuclease (SNase)

Oral delivery of SNase via engineered *Lactococcus lactis* degrades intestinal NETs, improves barrier integrity, and delays T1D onset in non-obese diabetic mice.^228^ This microbiota-targeted strategy is conceptually appealing, but the long-term safety of reshaping intestinal NET dynamics remains unclear.

### Pharmacological inhibition of NET formation

Small-molecule agents that intercept key signaling nodes governing NET formation, together with drugs that neutralize upstream NETogenic triggers, are emerging as tractable therapeutic avenues.

#### PAD4 inhibitors

The pan-PAD inhibitor Cl-amidine delays T1D onset, lowers autoantibody titers and reinforces gut barrier integrity in non-obese diabetic mice by selectively blocking NETosis.^229^ Despite compelling preclinical data, the therapeutic window for sustained PAD4 modulation—sufficient to curb autoimmunity yet permissive for host defense—remains undefined.

#### NLRP3 inflammasome inhibitors

Disulfiram, repurposed from alcoholism therapy, inhibits NLRP3/caspase-1/gasdermin D-mediated NETosis. In diabetic wound models, it accelerates healing, particularly when reformulated into nanovesicle hydrogels that enhance local delivery, promote macrophage M2 polarization, and limit systemic exposure.^230^^,^^231^ Nevertheless, dose, route, and exposure-response relationships for local formulations require rigorous clinical evaluation to ensure an acceptable risk-benefit profile.

#### PKCβ inhibitors

Ruboxistaurin, a selective PKCβ inhibitor, accelerates diabetic wound closure by promoting angiogenesis and restraining NETosis.^232^ This highlights the therapeutic potential of modulating diabetes-upregulated kinases, particularly in retinopathy and chronic wounds, where PKCβ hyperactivation drives tissue injury.

#### Anti-virulence strategies

Neutralization of *Staphylococcus aureus* α-toxin with monoclonal antibodies suppresses toxin-driven pathological NETosis, preserves tissue integrity and accelerates healing in infected diabetic wounds^233^— an approach that de-escalates pathogen fitness while maintaining immune competence.

#### Gonadotropin-releasing hormone (GnRH) signaling blockade

GnRH receptors on neutrophils augment PAD4-dependent histone citrullination. GnRH antagonists suppress NET formation and improve wound healing in diabetic models, with upregulated GnRH receptor expression in human DFUs underscoring translational relevance.^234^

#### Hydrogen sulfide (H_2_S) donors

H_2_S donors (e.g., Na_2_S) scavenge ROS, thereby blocking the ROS/MAPK (ERK/p38) pathway, reducing PAD4 induction and suppressing NETosis to facilitate diabetic wound repair.^235^

#### NF-κB inhibitors

The selective NF-κB inhibitor Ro 106-9920 downregulates PAD4 by blocking NF-κB nuclear translocation and interrupts the pro-inflammatory NET-macrophage NLRP3 feedback loop, reducing inflammation and restoring reparative cell function in diabetic wounds.^236^

Collectively, these pharmacological approaches targeting distinct NETogenic signaling nodes have been validated across multiple independent studies, underscoring the reproducibility of NET inhibition as a therapeutic strategy in diabetic complications.

### Immunomodulation and natural compounds

Beyond direct NET blockade, modulating the immune microenvironment provides an indirect means to temper neutrophil hyperactivity.

#### Micronutrient supplementation

Vitamin D_3_ suppresses PMA-induced NET formation in *ex vivo* neutrophils from patients with T2D,^119^ hinting at diet-based adjunctive strategies, though clinical validation remains needed.

#### Immunomodulatory antibiotics

In glycaemically well-controlled T2D, clarithromycin exerts context-dependent immunomodulation: rather than abolishing NET formation, it enhances NET antimicrobial and pro-healing potential by increasing LL-37 peptide loading, thereby promoting fibroblast migration and collagen deposition.^237^ Prudent use is advised, given potential QT prolongation and antimicrobial resistance.

#### Natural compounds

Preclinical studies implicate baicalin, costunolide, and thonningianin A—the latter benefiting from chitosan nanoparticle hydrogels—in mitigating NET-driven tissue injury via barrier protection, anti-thrombo-inflammatory activity, and M1-to-M2 macrophage repolarization.^115^^,^^238^^,^^239^ However, low oral bioavailability and formulation dependence remain translational hurdles.

#### Probiotics

Topical *Lactiplantibacillus plantarum* improves healing of chronic DFUs by reshaping the microbiota, enhancing angiogenesis and modulating neutrophil responses,^240^ suggesting a safe adjunctive route to indirectly influence NET activity.

### Advanced biomaterials and nano-therapeutics

Nanotechnology and engineered biomaterials are transforming the delivery and control of NET-targeted therapies, especially for chronic diabetic wounds.

#### Nanoparticle systems

Potassium-doped MnO_2_ nanoparticles recalibrate neutrophil calcium signaling, restore phagocytic competence, and improve healing in MRSA-infected diabetic wounds.^241^ DNase I-loaded vanadium carbide MXene nanoregulators redirect neutrophils from NETosis toward phagocytosis, disrupt biofilms, and clear infection.^242^ These platforms exemplify a shift from simple inhibition to the functional re-education of neutrophils, but comprehensive biodistribution, biodegradation, immunogenicity, and inflammasome-activation profiling is a prerequisite for first-in-human translation.

#### Engineered hydrogels

Multifunctional hydrogels provide sustained local drug delivery and microenvironment modulation. Examples include PAD4 inhibitor (TDFA)–loaded gels that suppress NETosis and promote macrophage polarization^243^; programmable hydrogels that sequentially release PDGF-BB and DNase I for synergistic cell recruitment and NET clearance^244^; and zwitterionic hydrogels that dampen inflammation while fostering regeneration.^245^ NET-scavenging hydrogel microspheres further neutralize NET components without releasing trapped pathogens.^246^ Manufacturing complexity, cost-of-goods, quality-by-design constraints, and regulatory validation in clinically relevant chronic-wound endpoints remain major barriers.

### Biological and cell-derived therapeutics

Cell-derived vesicles and endogenous proteins offer multi-target immunomodulation with innate biocompatibility.

#### Stem cell-derived extracellular vesicles (EVs)

Mesenchymal stem cell EVs promote angiogenesis and accelerate wound repair by preventing NET-induced ferroptosis in endothelial cells.^247^ Hypoxia-preconditioned small EVs deliver miR-17-5p to suppress the TLR4/ROS/MAPK axis, reduce NETosis, and alleviate fibroblast ER stress.^248^

#### Platelet-rich plasma exosomes (PRP-Exos)

Platelet-rich plasma exosomes (PRP-Exos) transfer miR-26b-5p to inhibit MMP-8–dependent NET formation, enhancing diabetic wound healing.^249^ However, scalable manufacturing, potency assays, and long-term stability remain unresolved for clinical translation.

#### Endogenous immunoregulatory proteins

Milk fat globule-epidermal growth factor 8 (MFG-E8) interrupts the NLRP3 inflammasome-NET positive feedback loop by suppressing IL-1β-driven NETosis and blocking NLRP3 activation via integrin β3–P2X7 interactions on macrophages. Recombinant MFG-E8 reduces inflammation, promotes angiogenesis, and accelerates diabetic wound healing.^250^

These cell-derived and endogenous biological therapies have been reported by multiple independent groups to exert coordinated immunomodulatory effects on NETs and tissue repair, highlighting their translational potential in diabetes-associated complications.

### Challenges and future directions

Despite robust preclinical progress, several barriers continue to limit clinical translation. The first is biological: NETs are integral to antimicrobial defense, so prolonged or systemic inhibition may predispose patients to bacterial or fungal infection.^251^ This concern is especially relevant in diabetes, where infection susceptibility is already increased. For this reason, localized, time-limited, or on-demand strategies, such as topical DNase-containing hydrogels, biomaterial depots, or short treatment windows during inflammatory flares, are likely to offer a safer early route to clinical testing.

A second barrier is disease heterogeneity. A therapeutic concept that is logical in a superficial chronic wound may not be appropriate for DKD, retinal microangiopathy, or pregnancy-associated disease. Translational development, therefore, needs to be phenotype-specific: topical nucleases, hydrogels, and EV-based approaches are best matched to DFUs; PKCbeta-, ROS-, or inflammation-modulating strategies may be more relevant in vascular and retinal injury; and nephropathy will probably require integration with established renoprotective drugs rather than NET targeting alone. Standardized biomarkers such as cfDNA, MPO-DNA complexes, and CitH3 should be used not merely as descriptive readouts but as tools for patient selection, pharmacodynamic monitoring, and adaptive dosing.^252^

Future work should define when NETs act as drivers, amplifiers, or bystanders across different stages of diabetic complications and should prioritize technologies that can resolve neutrophil heterogeneity *in situ*. Single-cell and spatial transcriptomics, multi-omics integration, and advanced *in vivo* imaging will be valuable for identifying the patients, tissues, and disease windows most amenable to intervention.^3^^,^^10^^,^^253^^,^^254^^,^^255^^,^^256^^,^^257^^,^^258^^,^^259^^,^^260^^,^^261^^,^^262^^,^^263^ In practical terms, the field will advance fastest by coupling mechanistic precision with clinically meaningful questions: which patients have a true NET-high phenotype, which compartments are safest to target, and what degree of NET modulation is sufficient to improve outcomes without compromising host defense.

## Conclusions

NETs occupy a context-dependent position at the intersection of metabolism, inflammation, thrombosis, and defective tissue repair in diabetes. Experimental and clinical evidence increasingly supports the view that dysregulated NET formation contributes to chronic wound persistence, retinal barrier failure, kidney injury, vascular thrombosis, and other diabetes-related complications, although the magnitude and meaning of NET activity vary by tissue, disease stage, and inflammatory context.

Clinically, the most realistic near-term application of this field is a precision strategy that combines standardized NET-associated biomarkers with complication-specific intervention. Local NET-degrading or NET-modulating therapies appear closest to translation for chronic wounds and accessible inflammatory lesions, whereas systemic approaches for renal, retinal, and vascular disease will require careful patient selection and preservation of innate immune function. Defining robust biomarkers, therapeutic windows, and tissue-specific response patterns will be essential for converting NET biology into practical benefit for patients with diabetes.

## Data and code availability

Data sharing is not applicable.

## Acknowledgments

Not applicable.

Funding: This research was funded by the Futian District of Shenzhen City Clinical Key Specialty Construction Fund (0998H).

## Author contributions

Conceptualization, literature search, L.L., Y.J.L, Z.Z.Z., and R.Y.L.; writing – original draft preparation, L.L. and Y.J.L; figure preparation, Z.Z.Z.; review and editing, Z.Z.Z. and B.H.; project administration, Z.Z.Z. and B.H.; all authors have read and agreed to the published version of the manuscript.

## Declaration of interests

The authors declare that they have no competing interests.
